# The adoption of the One Health approach to improve surveillance of venomous animal injury, vector-borne and zoonotic diseases in Foz do Iguaçu, Brazil

**DOI:** 10.1371/journal.pntd.0009109

**Published:** 2021-02-18

**Authors:** André de Souza Leandro, Renata Defante Lopes, Caroline Amaral Martins, Açucena Veleh Rivas, Isaac da Silva, Sandro Roberto Galvão, Rafael Maciel-de-Freitas

**Affiliations:** 1 Centro de Controle de Zoonoses, Secretaria Municipal de Saúde de Foz do Iguaçu, Paraná, Paraná, Brazil; 2 Laboratório de Transmissores de Hematozoários, Instituto Oswaldo Cruz, Fiocruz, Rio de Janeiro, Rio de Janeiro, Brazil; 3 Laboratório de Saúde Única, Centro de Medicina Tropical da Tríplice Fronteira, Foz do Iguaçu, Paraná, Brazil; 4 Instituto Nacional de Ciência e Tecnologia em Entomologia Molecular, Instituto de Bioquímica Médica, Universidade Federal do Rio de Janeiro, Rio de Janeiro, Brazil; Oregon State University College of Veterinary Medicine, UNITED STATES

## Abstract

Public health institutions with sectorized structure and low integration among field teams, old-fashioned practices such as paper-based storage system, and poorly qualified health agents have limited ability to conduct accurate surveillance and design effective timely interventions. Herein, we describe the steps taken by the Zoonosis Control Center of Foz do Iguaçu (CCZ-Foz) in the last 23 years to move from an archaic and sectorized structure to a modern and timely surveillance program embracing zoonotic diseases, venomous animal injuries, and vector-borne diseases epidemiology under the One Health approach. The full implementation of the One Health approach was based on 5 axes: (1) merging sectorized field teams; (2) adoption of digital solutions; (3) health agents empowerment and permanent capacitation; (4) social mobilization; and (5) active surveys. By doing so, notifications related to zoonotic diseases and venomous animals increased 10 and 21 times, respectively, with no impairment on arbovirus surveillance (major concern in the city). Open sources database (PostgreSQL) and software (QGis) are daily updated and create real-time maps to support timely decisions. The adoption of One Health approach increased preparedness for endemic diseases and reemerging and emerging threats such as Severe Acute Respiratory Syndrome Coronavirus 2 (SARS-CoV-2).

## Introduction

Brazil is recognized as hosting great taxonomic biodiversity within its biomes, which include forests, savannah, steppe, mangrove, and wetlands. The continuous human occupation and land use in biome ecotopes for either housing, farming, or livestock increases risk of spillover events and zoonotic diseases transmission in urban areas [1]. Due to its size, biotic and abiotic conditions, species distribution, and the richness of biome diversity, disease transmission risk in Brazil varies geographically and seasonally [2–4].

Ideally, public health institutions should be organized under principles of integration (to optimize available resources), personnel empowerment (favoring immediate decision by health agents on the ground), community engagement (to sensitize communities on good practices and support control efforts), and flexibility (to allocate health agents accordingly to the current emerging or seasonal public health treat) [5]. However, in most parts of Latin America, disease surveillance of tropical diseases is sectorized and independent from each other, i.e., there is an absence of integration among different teams in the Municipality. Furthermore, routine surveillance still relies on old-fashioned ineffective practices that jeopardize the adoption of timely decisions such as using paper-based notification systems to store field-gathered information and absence of continuous capacity-building of health agents [6].

Foz do Iguaçu (25° 30′ 58″ S 54° 35′ 07″ W) is a city located in the Paraná State, Brazil, more specifically on the Triple Border with Argentina and Paraguay, with approximately 250,000 inhabitants. Herein, we describe the steps taken by the Zoonosis Control Center of Foz do Iguaçu (CCZ-Foz) in the last 23 years to move from an archaic and sectorized structure to a modern and timely surveillance program embracing zoonotic diseases, venomous animal injuries, and vector-borne diseases epidemiology under the One Health approach [7]. We define the One Health approach as a synergic strategy for expanding interdisciplinary collaborations of health care for humans, animals, and the environment. The adopted One Health approach enhanced the preparedness for public health threats such as the current SARS-CoV-2 (COVID-19) pandemic [8].

### Zoonotic diseases

Zoonotic diseases are diseases transmitted from animals to humans. Therefore, the adoption of a One Health approach provides an ideal framework for understanding of direct and indirect interactions with potentially infected animal hosts.

Rabies virus (RABV) was detected for the first time in 1997 in 4 stray dogs and 2 domestic cats. Diagnostics were done through direct immunofluorescence assay from the Central Nervous System (CNS) and viral isolation in Swiss mice at Central Laboratory of Paraná State (LACEN-PR), located 630 km from Foz do Iguaçu. The first RABV cases triggered the CCZ-Foz creation aiming to suppress RABV outbreak in dogs and cats. Unusual aggressive behavior of dogs and cats started to be registered as an early sign of RABV. Animals were captured, removed to city shelters, and euthanized if aggressive behavior persisted and no claims for them was done by local citizens. In the 2000s, extensive RABV vaccination campaigns based on house-to-house surveys were adopted to immunize domestic cats and dogs and reached an estimated 55,000 animals per year. Most likely due to the comprehensive vaccination campaigns, the last RABV cases in dogs were documented in 2005. In 2001, CCZ-Foz stated to screen dead bats for RABV. In the 2001 to 2020 period, a total of 144 out of 3,480 bats were positive (4.13%). Human autochthonous cases of RABV were never registered in Foz do Iguaçu.

In 2008, the first case of human visceral leishmaniasis was recorded. Local transmission involves the sandfly *Lutzomyia longipalpis* infected with *Leishmania infantum* (class Kinetoplastea). Previous reports revealed the citywide distribution of *Lu*. *longipalpis* and a prevalence on dogs of 23.8% [9]. Injured dogs were taken by their owners or zoonotic diseases agents to the CCZ-Foz for screening by immunochromatography with further confirmation at LACEN-PR. From 2008 to 2020, a total of 24 cases of human leishmaniasis were reported, and between 2012 and 2020, a total of 4,426 cases of leishmaniasis in dogs were recorded in the city. Due to the observed disproportion of incidence in dogs and humans (185 times greater in the former), we classified leishmaniasis in Foz do Iguaçu as an infectious disease that occasionally jump from dogs to humans with the aid of *L*. *longipalpis*.

From 1997 to 2014, when the One Health approach was formally adopted, zoonotic diseases had an average of 10 health agents exclusively dedicated to zoonotic diseases-related issues.

### Vector-borne diseases

Vector-borne diseases are the diseases transmitted through the bite of arthropod vectors. Thus, the adoption of a One Health approach allows for inclusion of broader interacting factors including landscape, land use and management (e.g., discard of plastic containers, piped water availability, etc.), and social and climatic factors that drive disease transmission patterns. As with many Brazilian cities, vector-borne diseases are of utmost public health importance due to its high incidence rates. Among those, the mosquito-borne dengue virus (DENV) causes disease with high morbidity and mortality in the region. Therefore, historically, most public health resources from the 3 levels of government (municipality, state, and federal) are allocated to hire personnel, purchase of insecticides, equipment maintenance, and media engagement campaigns on radio, TV, and internet. From 1998 to 2014, the CCZ-Foz had an average of 100 to 120 health agents working on vector-borne diseases.

Mosquito vector control was originally conducted by health agents from the Brazilian Ministry of Health, with low participation of local public health managers in defining priorities and strategies. The control of malaria and arboviruses became CCZ-Foz responsibility as soon as it started operating in 1999. Surveilled differently from other diseases, malaria surveillance was based on the active survey of people with malaria symptoms with further transportation to the nearest health unit. Malaria cases in the region are caused by *Plasmodium vivax* (phylum Apicomplexa) after the biting of infected female mosquitoes *Anopheles darlingi* and *Anopheles albitarsis* species. Most of cases occur on the outskirts of areas flooded by the Paraná river dam, close to rivers on the surroundings of Iguazu National Park and the hydroelectric power plant of Itaipu [10]. After a few years of relative absence in 2000 to 2006, a malaria peak was reported in 2007. Since then, malaria cases have been regularly notified, and more than 2,500 cases have been reported in the city.

For arboviruses, Foz do Iguaçu experienced its first dengue outbreak in history in 1998. In 2002, the Brazilian Ministry of Health released the National Dengue Control Program, which elected Foz do Iguaçu as a priority city to receive Federal Investment on dengue to support vehicle and fogging equipment purchasing and insecticide application. Between 2002 and 2004, the city recorded the cocirculation of DENV-1, DENV-2, and DENV-3. In 2005, the CCZ-Foz adopted the *Aedes aegypti* Larval Index (LIRAa) as the official surveillance method, by which around 5% of houses are randomly selected to be inspected for breeding sites and immature mosquito collection [11]. However, the presence and abundance of larvae is frequently not related to adult density and consequently with disease transmission risk [12]. From 1999 to 2014, when the One Health approach was implemented in Foz do Iguaçu, the city recorded dengue outbreaks every 3 to 5 years most likely due to the absence of herd immunity in human population to a new serotype or to a serotype with no history of recent circulation [13].

### Injuries with venomous animals

Although the CCZ-Foz opened in 1999, the injuries caused by venomous animals started to be monitored only in 2006, when the first team was composed by 5 health agents to cover the entire city. The surveillance to venomous animals was triggered only after CCZ-Foz received the notification of an incident, i.e., an injury caused by a venomous animal a few days after a local citizen sought for medical assistance in a local health unit. By doing so, the team dealing with injuries by venomous animals was only reacting to incidents notified a few days earlier. Occasionally, by the time the health agents dealing with venomous animals arrived at the location where notification took place, no further action was required. From the notifications received by the venomous animals team, the majority of them was related to spiders (56.1%), bees (24.4%), and snake bites (7.4%).

One Health team implementation

The tipping point to reorganize health agents’ activities under the One Health framework came after a series of seminars with scientists, public health managers, health agents, and local decision-makers in Foz do Iguaçu. At that time, it became clear that a traditional approach relying on isolated and fragmented actions that overlook environmental factors and animal health would not be sufficient to attain better health for people. Notwithstanding, the path to promote this change started in the late 1990s but was implemented in 2014, after all levels of government, local social entities, and municipality stakeholders realized the relevance of adopting an integrated approach. An integrated One Health approach would promote a better understanding of the intrinsic complexity of disease transmission since it emphasizes the relatedness of human, animal, environmental health, and the importance of transdisciplinary efforts. The full implementation of the One Health approach was based on 5 axes.

### Merging sectorized field teams

From 1999 to 2014, each of the field teams (zoonotic diseases, vector-borne diseases, and venomous animals) had a fixed composition of health agents. Before merging field teams into the One Health team, 86.4% of health agents were allocated to dengue-related activities [14]. Thus, at that time, investigations related with zoonotic diseases and venomous animals were largely underrepresented. For instance, between 2010 and 2013, health agents of the vector-borne diseases team visited an average of 31,970 dwellings per month; meanwhile, zoonotic diseases and venomous animals teams visited a monthly average of 80 and 369 houses, respectively.

The reorganization of health agents’ activities was associated with restructuring the CCZ organizational chart. Hiring new health agents required broad training in One Health applications versus sector-specific activities (the usual practice up to 2013). The adoption of the One Health approach increased the number of visits to zoonotic diseases and venomous animal events by 10 and 21 times, respectively. The number of visits related to vector-borne diseases increased by 15%. An additional benefit from the One Health strategy was that after oral consent by the householder, a single health agent was able to conduct a detailed and a comprehensive survey of the premise. Another visit by that health agent will only occur on the next month, unless the health agent identifies the need to return in less than 30 days, i.e., to follow up notifications from the previous visit, e.g., checking whether mosquito containers were eliminated, premise refurbishment, etc.

### Adoption of digital surveillance approaches

Part of the reorganization of CCZ-Foz was only achieved in 2015. By that time, storing field notification sheets became unfeasible, and digitizing information was adopted to reduce the time required for processing field-gathered data.

Since 2015, all activities conducted by the One Health Team have been added to an open-source database (PostgreSQL), which is integrated with a free and open-source geographic information system (QGIS). This integration allows the production of daily-updated maps to inform local decision-makers the precise location of each notification. For example, specific risk maps can be created based on occurrences such as RABV-positive bats, or which city block has the highest *Ae*. *aegypti* infestation index and where domestic dogs with visceral leishmaniasis were collected. Such information is critical to increase preparedness of the city by providing guidelines for specific, effective, and timely response.

### Health agent empowerment and continuous training

Newly hired health agents were subject of a training course of 80 h (40 h theoretical and 40 h practical) focused on the scope of CCZ-Foz and the One Health approach. For instance, focus was placed on public health policies, natural history, and epidemiology of locally transmitted diseases, environmental ecology, open software used in CCZ-Foz routine, and the One Health approach. As soon as emerging and reemerging diseases became a critical issue in Foz, like the arrival of Zika and chikungunya in 2015 and the COVID-19 outbreak in 2020, new rounds of training were scheduled to update health agents.

Therefore, with close supervision in the first months, health agents were trained and empowered to conduct best practices simultaneously on zoonotic diseases, vector-borne diseases, and injuries with venomous animals. Health agents were capable of understanding the transmission risks and epidemiology of each individual disease and conducting comprehensive surveys to meet the adequate disease mitigation activity. After the One Health approach was implemented, each householder received a single detailed visit by a trained health agent. This triggered a win-win situation, in which residents started to feel better cared by CCZ-Foz and health agents were better received by residents.

### Social mobilization

The CCZ-Foz fostered the creation of a social mobilization and education team that is responsible for the production of educational material with an integrated approach and an emphasis on One Health with topics related to zoonotic diseases, vector-borne diseases, and injuries with venomous animals. The prevention and control of zoonotic diseases, vector-borne diseases, and venomous animal injury was included as a mandatory theme in the local school curriculum. Public and private school teachers were trained to work as amplifiers in their respective classrooms, addressing One Health-related themes throughout the school year. Since the adoption of One Health in 2014, there has been an estimated number of 55,000 students from 6 to 16 years old that were sensitized. Scaled models, lectures, pamphlets, and science fairs have been used as dissemination tools. Annually, an average of 15,000 people has been directly reached by the permanent team of 10 CCZ employees in activities related to schools, residents' associations, and religious entities. The social mobilization team worked in tandem with the municipal government's social communication sector, which disseminates information via radio, television, newspapers, and the internet to achieve better social engagement for public health efforts [5].

### Active surveillance

Before 2014, active surveillance was performed only in malaria since health agents were accustomed to visit the isolated communities on the banks of Paraná river to direct those with malaria symptoms toward the closest health unit. After merging field teams and empowering health agents to conduct a comprehensive visit, surveillance to all other diseases became naturally active. The action following the perception of a public health notification under the scope of One Health was immediate. As health agents visualized the occurrence, an urgent action was elicited, i.e., the time required to respond to the reported notification dropped dramatically.

## Results and challenges of One Health approach

**Fig 1:**
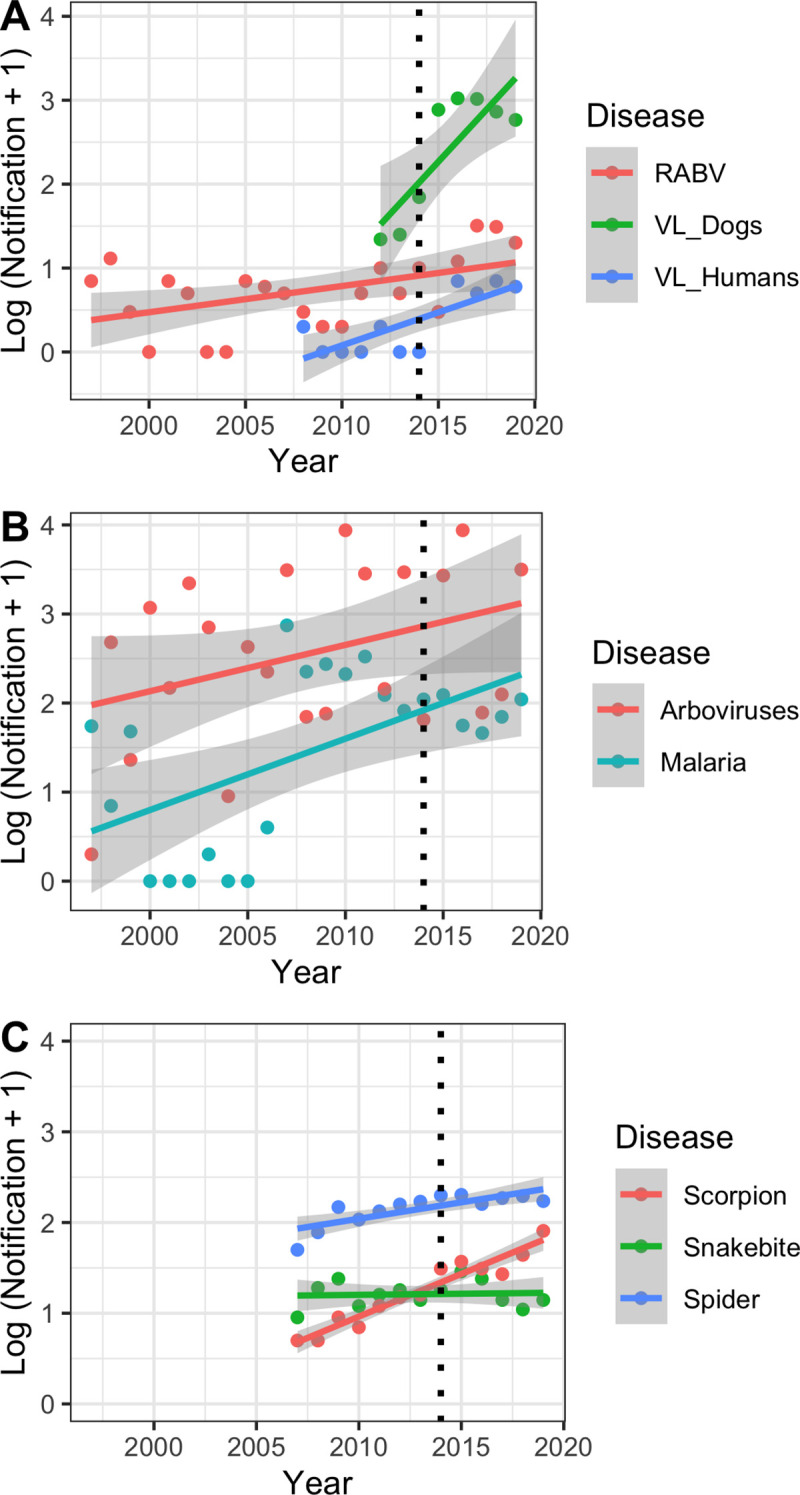
Disease notification in Foz do Iguaçu from 1997 to 2019. (A) Zoonotic diseases. RABV relates to the sum of rabies notifications in dogs, cats, and bats (since 2005, only bats were found with RABV). (B) Vector-borne diseases. Arboviruses relate to the sum of dengue, Zika, and chikungunya cases; the last two arrived in Foz do Iguaçu only in 2015. (C) Injuries of venomous animals. Incidents related to bees and caterpillars were omitted from the graphs due to their low occurrence. We transformed the raw data by adding +1 to avoid issues with 0 cases.

The expectation of reducing disease notification at local level after empowering health agents revealed to be opposite of our original expectation. The comparison of the annual confirmed notification averages between 1997 and 2013 (pre-One Health) and 2014 and 2020 (post-One Health) highlighted that all diseases covered by CCZ-Foz do Iguaçu have an average increase in notifications after the adoption of the One Health approach (Fig 1). RABV reports increased 4.37 times, although all positive reports since 2005 were only in bats. Mass vaccination program based on a house-to-house survey on more than 90% of domesticated dogs and cats most likely controlled RABV. Arboviruses have an increased incidence of 1.92 times, and Zika and chikungunya were first recorded in the city in 2015. Visceral leishmaniasis increased 31.4 and 11.5 times in dogs and humans, respectively. Snake bite and injuries with spiders and scorpions increased 1.15, 1.54, and 4.25 times, respectively (Fig 1). Rather than observing a decrease in disease transmission, the presence of around 140 to 150 trained and empowered health agents in the One Health team helped gathering more robust data regarding each of the diseases under the scope of CCZ-Foz do Iguaçu.

*Ae*. *aegypti* surveillance was supplemented by installing almost 3,500 mosquito traps (Adultraps), one for every 25 houses, covering the entire city. Adultraps attract more often *Ae*. *aegypti* females seeking for a breeding site to lay eggs [15]. Those traps were inspected every month and provided valuable information regarding the areas of the city with higher adult mosquito population. Infestation indexes based on the presence and abundance of adult *Ae*. *aegypti* in the mosquito traps are under development.

The rationale and flexibility that underlies the One Health approach allowed health agents to efficiently assist and surveil areas during the initial SARS-Cov-2 arrival in Foz do Iguaçu. The flexible structure of the One Health team was adjusted to provide support for COVID-19. Health agents provided support on the territory and partnered with local nurses to conduct active surveys and interview householders and referred those with COVID-19 symptoms to the closest health unit. Due to the focus on COVID-19, the number of houses visited by the One Health team decreased by 13.68%. As a protective measure for health agents and householders to constrain COVID-19 transmission, a Federal decree limited the inspection of health agents to the peridomestic area of dwellings. Since *Ae*. *aegypti* mosquitoes are highly adapted to human dwellings, blood-feeding mainly on humans, resting inside domestic environment, and laying eggs in man-made containers, most likely the infestation will increase as long as COVID-19 restrictions last [16]. Those points likely contributed to Foz do Iguaçu experiencing in 2020 the most intense dengue outbreak in history with 17,694 confirmed cases, the majority of them with DENV-2.

In conclusion, by merging the zoonotic diseases, vector-borne diseases, and venomous animals teams into a highly skilled and empowered team under the scope of the One Health approach, CCZ-Foz increased agents’ critical eye. As a consequence, the capacity of health agents was better equipped to screen houses for transmission risk diseases covered by CCZ-Foz, especially diseases which had incidence rates previously underestimated. The adoption of open-source database and software provided the modernization of information processing and rapid visualization of disease notification by maps, favoring local decision-makers to elicit rapid response to mitigate transmission. Most likely, the adoption of the 5-axis described herein will be able to enhance the local knowledge regarding disease transmission.
